# Immunomodulation of Avian Dendritic Cells under the Induction of Prebiotics

**DOI:** 10.3390/ani10040698

**Published:** 2020-04-17

**Authors:** Vladimir Zmrhal, Petr Slama

**Affiliations:** Department of Animal Morphology, Physiology and Genetics, Faculty of AgriSciences, Mendel University in Brno, Zemedelska 1, 613 00 Brno, Czech Republic; xzmrhal1@node.mendelu.cz

**Keywords:** avian dendritic cells, prebiotic, pattern recognition receptors, antigen-presenting cell, chicken

## Abstract

**Simple Summary:**

Dendritic cells recognize pathogen-associated molecular patterns in chicken intestines and are part of the initial immune response. The immunoregulatory properties of prebiotics acting in several ways in poultry have been known for many years. According to their function, dendritic cells should play an indispensable role in the proven effects of prebiotics on the intestinal immune system, such as through activation of T and B cells and cytokine production. Currently, there are no studies concerning direct interactions in poultry between non-digestible feed components and dendritic cells. Whereas most in vitro experiments with chicken dendritic cells have studied their interactions with pathogens, in vitro studies are now needed to determine the impacts of prebiotics on the gastrointestinal dendritic cells themselves. The present lack of information in this area limits the development of effective feed additives for poultry production. The main purpose of this review is to explore ideas regarding potential mechanisms by which dendritic cells might harmonize the immune response after prebiotic supplementation and thereby provide a basis for future studies.

**Abstract:**

Although the immunomodulatory properties of prebiotics were demonstrated many years ago in poultry, not all mechanisms of action are yet clear. Dendritic cells (DCs) are the main antigen-presenting cells orchestrating the immune response in the chicken gastrointestinal tract, and they are the first line of defense in the immune response. Despite the crucial role of DCs in prebiotic immunomodulatory properties, information is lacking about interaction between prebiotics and DCs in an avian model. Mannan-oligosaccharides, β-glucans, fructooligosaccharides, and chitosan-oligosaccharides are the main groups of prebiotics having immunomodulatory properties. Because pathogen-associated molecular patterns on these prebiotics are recognized by many receptors of DCs, prebiotics can mimic activation of DCs by pathogens. Short-chain fatty acids are products of prebiotic fermentation by microbiota, and their anti-inflammatory properties have also been demonstrated in DCs. This review summarizes current knowledge about avian DCs in the gastrointestinal tract, and for the first-time, their role in the immunomodulatory properties of prebiotics within an avian model.

## 1. Introduction

Gibson and Roberfroid [1] described prebiotics for the first time in their 1995 paper as “a non-digestible food ingredient that beneficially affects the host by selectively stimulating the growth and/or activity of one or a limited number of bacteria in the colon, and thus improves host health.” This definition is incomplete, however, because it does not mention the immunomodulatory properties of prebiotics. Prebiotics act in several ways to eliminate pathogen colonization in the gastrointestinal tract (GIT). Some prebiotics bind to type 1 fimbriae of pathogenic bacteria and inhibit their colonization. Additionally, prebiotics cause higher production of mucin by increasing the number of goblet cells. After prebiotic treatment, beneficial microbiota can produce organic acids and bacteriocins that subsequently prevent colonization by pathogenic bacteria [2]. Prebiotics can directly affect the immune response by pathogen-associated molecular patterns (PAMPs). PAMPs are recognized by pattern recognition receptors (PRRs) expressed on antigen-presenting cells of the immune system [3]. Dendritic cells (DCs) are the main antigen-presenting cells, and they are universal immune cells responsible for several functions. These cells are capable of endocytosis, exocytosis, antigen processing and presentation, and cytokine production as well as activating innate immune responses and specific acquired immunity [4]. DCs are located in tissues that are in contact with the external environment. The GIT is the part of the chicken body interior most exposed to the external environment. DCs are well equipped with several types of receptors to distinguish PAMPs in the GIT, and after their activation, DCs migrate to the lymphoid tissues where they interact with T cells in diffuse lymphoid tissues and with B cells in germinal centers. In the intestine, stimulation of DCs is required to activate the host-protective immune response to infections [5].

Despite the quintessential role of DCs in prebiotic-induced immunomodulation in chickens, there have been few studies about interactions of avian DCs with prebiotics. Experiments using human or mouse models, meanwhile, have shown direct and indirect effects of prebiotics on DCs. In the first part of this review, we summarize our knowledge about intestinal DCs. In the second part, we focus on the potential role of DCs in prebiotic-induced immunomodulatory processes.

## 2. Dendritic Cells Are the Most Important and Effective Antigen-Presenting Cells 

As major antigen-presenting cells, DCs play crucial role in the immune response because they can present antigens to naive T cells and B cells. Although, this ability has been also proven in macrophages [6]. The antigen-presenting function of B cells is apparently minor because Beal et al. [7] have proven the normal T cell response in bursectomized chickens treated with *Salmonella enterica* serovar Typhimurium. DCs and macrophages are derived from bone marrow hematopoietic stem cell precursors expressing CD45^+^ [4]. The antigen-presenting function in macrophages is complementary because these cells are well equipped to destroy entering pathogens. DCs, on the other hand, are crucial in activating the adaptive immune response [8]. Antigen presentation consists of antigen intake, subsequent processing of antigen in lysosomes, then presentation of antigen peptides by major histocompatibility complex (MHC) class I and class II molecules to naive T cells [9]. During this process, antigen peptides in DCs are not fully destroyed in lysosomes because DCs have much lower levels of lysosomal proteases and are capable to alkalizate the environment in lysosomal units [10,11]. Proteolytic activity in macrophage lysosomes is much greater, and that limits their antigen-presenting function [11]. Based on the phagosome acidification level, DCs and macrophages can be distinguished by surface markers. De Geus et al. [12] characterized cells in chicken lungs after uptake of fluorescently labelled beads coated with either lipopolysaccharide (LPS) or inactivated avian influenza virus in relation to phagosome acidification. They observed higher acidification expressed as lower pH in CD40^+^, CD11^+^, and KUL01^+^ cells and identified these as macrophages [13]. Cells that did not decrease pH in their lysosomal units expressed typical DC activation markers on their surfaces such as MHC II^+^ and CD80^+^ [12].

## 3. Avian Dendritic Cells

Avian DCs were described for the first time by Olah and Glick as secretory cells in the medulla within the bursa of Fabricius [14] and subsequently in the germinal centers of cecal tonsils in chickens [15]. DC subtypes are derived from hematopoietic stem cell progenitors, but Gomez Perdiguero et al. [16] have proven that epidermal DCs could be derived from yolk-sac-derived erythro-myeloid progenitors. Development of DCs through ontogenic pathways was reviewed by Merad et al. [17], and ontogenesis with a focus on chicken dendritic cells was summarized by Nagy et al. [4]. For contact with antigens, it is necessary for DCs to be directly located in almost all tissues of the chicken body, including mucosal surfaces, interstitial tissues, skin epidermis, peripheral blood, and non-lymphoid tissue, but the lymphatic tissue is crucial for DC migration and antigen-presenting functions [18]. In recent years, there have been increased numbers of in vitro studies using chicken DCs treated with viral diseases such as infectious bursal disease [19,20], Newcastle disease [21], and avian influenza [22], or with bacterial diseases like *Salmonella* [23,24]. Numerous specific markers have been described for in vitro characterization of chicken DCs. Macrophage progenitors, monocytes, and DCs express colony-stimulating factor 1 receptor (CSF1R), which is a target for granulocyte macrophage colony-stimulating factor necessary for differentiation and proliferation [25]. Constitutive expression of CSF1R is a way to distinguish monocytes from other myeloid cells, including heterophils and thrombocytes [26]. Immature bone marrow-derived DCs show expression of MHC class II^+^, CD11c^+^, CD40^+^, CD11^+^, CD86^+^, CD83^−^, and DEC205^−^ [27]. Chemokine receptor CCR6 is expressed on immature DCs. After stimulation of CCR6, DCs migrate to sites of antigen entry. After stimulation by antigen, DCs mature and acquire the ability to present antigen to T cells. Maturation is represented by up-regulation of CCR7 and down-regulation of CCR6 [28]. Stimulation by LPS causes great increase in expressions of CD40, CD11, CD86 [27], CD83 [29], and DEC205 [30]. Kalaiyarasu et al. [31] have suggested that induction of nitric oxide synthase may also be used as a maturation marker for chicken DCs.

## 4. Dendritic Cells in the Chicken Gastrointestinal Tract

The GIT has the most extensively exposed surface within the body, and DCs play the most important role in distinguishing between the harmless particles and potential pathogenic microorganisms that are continuously passing through the GIT [32]. Dendritic cells have been described in clusters of gastrointestinal-associated lymphoid tissue (GALT). Esophageal tonsil consists of mucosa-associated tissue around the entrance of the proventriculus. Dendritic cells in esophageal tonsils are exposed to undigested feed containing pathogens. For this reason, these DCs can play a crucial role in successful application of oral vaccines and nutritional immunomodulators such as prebiotics. Lymphoepithelial tissue includes vimentin^+^, MHC^+^, and 74.3^+^ stellate-shaped cells recognized as a DC. Typically, follicular dendritic cells (FDCs) in germinal centers are covered by an electron-dense substance containing IgG and express vimentin and 74.3 [33]. The same FDC has been described in germinal centers of pyloric tonsils located at the beginning of the duodenum [34]. In Peyer’s patches, ellipsoid-associated cells have been described that are proposed to be precursor cells of interdigitating dendritic cells (IDCs) and FDCs, depending on the location in the lymphoid tissue [35]. Ellipsoid-associated cells have been found in spleens within the antigen-trapping zone of the blood–spleen barrier. After their stimulation by antigen, these cells differentiate into IDCs, induce germinal center formation by recruiting proliferating B cells, and subsequently differentiate into FDCs [36,37,38]. The observed presence of ellipsoid-associated cells in spleen suggests that these cells migrate from spleen to the GIT, where they serve as DC precursors [39]. After *Eimeria* infection in chickens, IDCs also have been found, together with FDC-like cells, interacting with parasite in cecal tonsils. However, isolated CD45^−^ and MHC II^−^ cell populations are unique within the functional properties of FDCs, such as stimulation of IgG production by allogeneic B cells in an MHC-unrestricted manner. Another type of cell consists of CD45^+^, MHC I^+^, and MHC II^+^ IDCs with abilities to induce proliferation of naive allogeneic CD4^+^ cells and augment the secretion of IFN-γ by allogeneic cells. It is noteworthy that the FDCs did not express the peripheral blood mononuclear cells marker CD45. This was in contrast to other studies that proved CD45 expression of FDCs [4,5].

## 5. Avian Dendritic Cells Pattern Recognition Receptors and Their Ligands

### 5.1. Toll-Like Receptors

PRRs play an important role in the innate immune response because it is through PRR antigen-presenting cells, such as DCs, that pathogens are recognized because of the occurrence of PAMPs on pathogen surfaces. Toll-like receptors (TLRs) are among these receptors. Immature DCs express a full set of TLRs, and after ligand binding maturing begins [40]. TLR ligands bind to TLRs, and this is the most common means of activation to become mature DCs [41]. To date, the identified TLRs in chickens are TLR 1A, TLR 1B, TLR 2A, TLR 2B, TLR 3, TLR 4, TLR 5, TLR 7, TLR 15, and TLR 21 [42]. Each TLR has a unique specificity to a different PAMP, so individual TLRs have limited responses to pathogens. The TLR family comprises a wide range of ligands from viruses, bacteria, fungi, and parasites and can ensure recognition for most known pathogens. LPS is the major component of Gram-negative bacteria, and most pathogens have this type of cell wall. TLR 4 is the main receptor for LPS, and CD14 is important for its function as required for the microbe-induced endocytosis of TLR 4. In DCs, this CD14-dependent endocytosis pathway is up-regulated upon exposure to inflammatory mediators [43,44]. Similarly, TLR 2B has affinity for LPS, lipopeptide, and glycopeptide [45]. TLR 5 recognizes flagellin, a monomeric subunit occurring in flagella of Gram-positive and Gram-negative bacteria [46]. TLR 3, TLR 7, and TLR 8 ensure intracellular recognition of RNA and DNA, so their specificity is mainly to viral compounds [47]. TLR 15 is activated by fungal proteases and also by some proteases of bacterial origin [48]. TLR 21 recognizes a broad repertoire of synthesized cytosine–phosphate–guanine (CpG) DNA molecules and responds to bacterial chromosomal DNA. CpG is a known ligand of human TLR 9, which is absent in avian species [49,50]. Different ligands determine the localization of TLRs in DCs because recognition of bacterial antigens requires localization of TLR 1, TLR 2, TLR 4, and TLR 5 on the outer surfaces. On the other hand, TLR 3, TLR 7, and TLR 8, with their ability to distinguish viral nucleic acids, are located in intracellular endosomes and lysosomal compartments, thereby enabling them to determine pathogen nucleic acids [40].

#### Toll-Like Receptor Ligands

Various TLR ligands have been studied for vaccine adjuvant function. Cytosine–phosphate–guanine oligonucleotides (CpG ODNs) bind to TLR 21, and CpG ODN treatment has been proven to enhance vaccine immunogenicity against *Salmonella Typhimurium* [51], *Eimeria* [52], and low pathogenic avian influenza subtype H9N2 [53]. Furthermore, CpG ODN and LPS can delay the progression of Marek’s disease, perhaps because of higher specific antibody response [54]. TLR 4 ligands also have proven potential as vaccine adjuvants for in ovo vaccination, probably because avian influenza virus replication is reduced because of induction of IFN-γ stimulatory genes in chorioallantois membranes [55]. St. Paul et al. [56] studied the immunogenic effects of TLR 2 ligand Pam3CSK4 and flagellin on polarizing of T cells into Th1 or Th2 pathways. Both ligands stimulated the production of Th1-associated cytokines IFN-γ and IL-12 as well as Th2-associated cytokine IL-4 and induced strong direct immune response. Pam3CSK4, LPS, and CpG ODN also are able to induce much higher nitric oxide production in macrophages [57]. Pam3CSK4 and poly I:C (TLR 2 and TLR3 ligands, respectively) synergistically up-regulated IFN-ß, IFN-γ, IL-12, and IL-4, and they cross-inhibited IL-1ß, IL-10, and iNOS in peripheral blood mononuclear cells. This reduced the destruction of B cells as well as bursal damage caused by infectious bursal disease virus (IBDV) when used with a hot IBDV vaccine [58]. Targeting TLR 3 and TLR 21 by a combination of their ligands, double-stranded RNA and CpG ODN, respectively, caused a pro-inflammatory immune response in chicken monocytes by up-regulating IL-8, IL-1β, IL-6, and MIP-1β and promoting Th1-biased immune response to chicken monocytes. In later stages, up-regulation of IL-10 and IL-4 is indicated to be a self-regulatory mechanism for controlling excessive inflammation [59]. Recent results have shown that using TLR 4 and TLR 21 ligands and their combination resulted in more sensitive innate immune responses in macrophages from birds 4 weeks old compared to birds 1 week old. These results suggest a need to reconsider the use of vaccine adjuvants in very young birds [60].

### 5.2. Carbohydrate-Binding Proteins

Carbohydrate-binding proteins (lectins) can recognize glycan structures on pathogen surfaces. They comprise various subtypes, such as galectins, siglecs, and collectins. Among the collectins are mannose-binding lectin [61,62], surfactant protein A (SP-A), and the specific chicken collectins CL-1, CL-2, and CL-3 [63]. A calcium-dependent chicken lung lectin also has been identified in the chicken respiratory tract [64]. On DEC205^+^ chicken myeloid cells, DC-specific intercellular adhesion, molecule-3-grabbing non-integrin (DC-SIGN), and macrophage galactose binding lectin were observed [8]. The use of monoclonal antibodies against C-type lectin endocytic receptor DEC205 on DCs provides potential for improving the immune response due to enhanced processing and presentation of antigen via MHC II [65,66]. Mannose receptor (MR) is a well-described cell-bound receptor in macrophages, but chicken DCs also express MR [8,67]. The roles of MR include recognition of mannan structures in a wide range of bacteria and viruses [68] and, subsequently, processing and antigen presentation by antigen-presenting cells [69,70].

### 5.3. Nucleotide-Binding Oligomerization-Domain-Like Receptors

For antigen recognition to function appropriately, receptors must be located in the cytosol to detect intracellular PAMPs, such as nucleotide-binding oligomerization-domain-like receptors (NODs). Among the NODs are NOD-1, which is able to recognize γ-glutamyl diaminopimelic acid, and NOD-2, which recognizes muramyl dipeptide, both of which are breakdown products of peptidoglycan that commonly occur on many bacteria [71,72]. These receptors are involved in the creation of inflammasomes, which, after antigen catalyzing activation of immature pro-inflammatory cytokines IL-1β and IL-18, are able to mature IL-1β and IL-18 and subsequently activate inflammatory processes or cell death. Inflammasomes are named after the NOD-like receptors with which they are involved, so we distinguish the NLRP3, NLRP1, and NLRC4 inflammasomes [73]. NLRC5 is a positive regulator of IFN-α and IFN-ß expression [74] and a negative regulator of MHC I expression [75,76].

### 5.4. Retinoic-Acid-Inducible Gene I-Like Receptors

Another important family of receptors located in the cytosol and well equipped for virus PAMP recognition consists of retinoic-acid-inducible gene I-like receptors (RLRs). The most important viral RNA-recognizing members of this group are RIG-I and melanoma differentiation-associated protein 5 (MDA5), both of which are able to distinguish cytosolic triphosphates in single-stranded RNA and double-stranded RNA [77]. Both subtypes have the same adaptor protein, known as mitochondrial antiviral-signaling protein (MAVS), involved in the RLR-mediated signaling pathway [78]. MAVS depletion disrupts pro-inflammatory and anti-virus cytokines production that is promoted by virus infection, so MAVS has an indispensable role in innate anti-virus immunity [79]. In contrast to waterfowl, chickens lack RIG 1 [80]. RIG 1 plays a crucial role in the immune response against Newcastle disease by up-regulation of IFN-β and mRNA levels of IRF 3 and IFIT1, which has been proven in geese [81]. In cases of low pathogenic avian influenza, ducks can up-regulate RIG 1 and limit virus replication in lungs by robust up-regulation of IFN-β [82,83]. In chickens, the role of RIG 1 is taken over by MDA5, but MDA5 probably does not play a decisive role during the immune response to influenza virus. MDA5 function is highly dependent on influenza strain because different strains have different RNA complements that could interact differentially with MDA5 [84]. Furthermore, the MDA5 signaling pathway is inhibited by the viral non-structural protein 1 [85]. On the other hand, it also has been proven that MDA5 strongly stimulates INF-β after infection by avian influenza virus. Thus, it is not unambiguous that a higher sensitivity to avian influenza virus in chickens is related to an absence of RIG 1. In their recent review, Evseev and Magor [86] have summarized several other differences between ducks and chickens in relation to immunity against avian influenza.

## 6. Role of Dendritic Cells in Prebiotic-Induced Immunomodulation

DCs harmonize the immune response in chicken intestines by producing cytokines and stimulating other immune competent cells. Prebiotics are able to affect DC function in both direct and indirect ways and beneficially modulate the immune response [3].

### 6.1. Mannan Oligosaccharides

Mannan oligosaccharides (MOS) occupy the outer layer of yeast cell wall. Yeast cell wall glycoproteins contain 50–90% carbohydrates that are characterized as mannans. Mannans consist of D-mannose subunits covalently bonded with proteins by two linkages: O-linked oligosaccharides and N-linked oligosaccharides. The first linkage consists of five mannose residues O-glycosylated with threonine or serine. Second are N-linked oligosaccharides that are N-glycosylated [2]. DCs are well equipped by PRRs to recognize yeast cell wall structures, and some studies have suggested which receptors are involved in this process. Studies in avian models have proven greater expression of TLR 2 and TLR 4 in chickens fed MOS [87,88] or after in ovo administration [89]. TLR 4 together with the MD-2 molecule constitutes a known receptor for LPS from Gram-negative bacteria [45,90]. TLR 2 is a principal receptor for peptidoglycan recognition of both Gram-negative and Gram-positive bacteria [91]. In a study with human mononuclear cells and *Candida albicans*, Netea et al. [92] found several receptors to be able to target different parts of MOS molecules. First, TLR 4 binds O-linked oligosaccharides from the MOS structure. MR, together with DC-SIGN, recognizes N-linked mannans and stimulates production of pro-inflammatory cytokine IL-6 [93]. Alizadeh et al. [88] proved yeast cell wall recognition by MR because MR was highly expressed in the ileum and cecum of chickens fed yeast with MOS-rich cell walls. Notably, chickens treated with *Clostridium perfringens* and supplemented with MOS had significantly higher expression of TLR 2 and TLR 4 in the intestine compared to a group treated only with *Clostridium perfringens* [87]. Furthermore, Lu et al. [94] treated chickens only with *Clostridium perfringens* and showed no significant up-regulation or down-regulation of TLR 2 and TLR 4. This suggests an ability of MOS to strengthen the recognition of bacteria by DCs. Stronger stimulation of TLR 2 and TLR 4 by MOS leads to activation of an MyD88-dependent pathway and production of pro-inflammatory cytokines such as IL-12 or TNF-α. After MOS supplementation, significant up-regulation of IL-12 and IFN-γ in ileum and cecal tonsils of chickens was determined [87]. IL-12 is produced by DCs and stimulates development of IFN-γ-producing Th1 cells from naïve CD4^+^ T cells [94,95,96]. As a pro-inflammatory cytokine, TNF-α promotes inflammatory processes. In mice, however, it has been proven that production of TNF-α by immature DCs is necessary for the development of IL-10-producing T cells. This points to TNF-α’s having an immune regulatory nature [97]. Immune regulatory mechanisms could explain the higher production of IL-10 in intestines of chickens supplemented with MOS [87,88]. DCs produce IL-10 in order to mitigate excessive inflammation. Rajput et al. [98] stimulated chicken bone marrow-derived dendritic cells (BMDDCs) with MOS-covered *Saccharomyces boulardii* (SB) and found significantly greater production of IL-10, thus confirming a self-regulatory mechanism in DCs. Chicken BMDDCs pulsed with SB under in vitro conditions showed responses similar to those observed in previous studies with MOS-treated chickens. Scanning electron microscopy revealed attachment and various engulfing stages of SB in chicken BMDDCs at different time intervals. Transmission electron microscopy of SB-pulsed chicken BMDDCs found lower levels of SB internalization with BMDDCs 3 h post-stimulation. On the other hand, 6 and 12 h post-stimulation, most SB particles were engulfed by DCs and showed various stages of degradation. Determination of MHC II and costimulatory molecules CD40, CD80, and CD86 expression was consistent with the electron microscopy findings because they increased with time post-stimulation. BMDDCs showed greater expression of TLR 2 and TLR 4 with up-regulation of the MyD88 pathway and NF-kB activation 6 h post-stimulation. Expression of pro-inflammatory cytokines was almost the same as that in the untreated control group. On the other hand, expression of anti-inflammatory cytokines TGF-β and IL-10 also was up-regulated [99]. Determination of anti-inflammatory cytokines expression was made 12 h post-stimulation, by which time self-regulatory mechanisms of DC were up-regulated [98].

Signaling through MR on DCs can support MR-mediated internalization and favor cross-presentation through MHC I in addition to MHCII-mediated presentation of antigens [68]. CD8^+^ T cells activated after cross-presentation play an important role in specific responses to cell-associated viral antigens [100].

Tohid et al. [101] evaluated antibody titers in chickens supplemented with MOS and vaccinated against avian influenza virus (AIV). An experimental group of chickens fed MOS had significantly higher production of AIV antibody titers in comparison to a vaccinated control group. DCs can play an important role in mechanisms contributing to much better antibody responses against AIV. It has been proven that chicken DCs recognize glycans (terminal Galα1-3Gal-R, chitotriose, Fucα1-2Galβ1-4GlcNAc-R) from AIV by MR and DEC205 receptors [8]. PAMP from MOS can be recognized by DCs located among epithelial cells in Lieberkühn crypts along the intestine [5]. Goblet cells play an important role in antigen delivery because they work as carriers delivering low molecular weight antigens from the intestinal lumen to DCs [102]. Subsequently, the activated T cells and DCs migrate to germinal centers of gastrointestinal-associated lymphoid tissue, where they activate B cells and stimulate production of plasmatic and memory B cells. Subsequently, B cells migrate through the lamina propria to the intestinal villi, where DCs and T cells induce in B cells high levels of IgA production. Subsequently, a part of B cells migrates through the bloodstream to the germinal centers of the spleen, where they activate other B cells and stimulate systemic antibody protection [103]. Newcastle disease virus, like other viruses, contains sialic acid-dependent glycans that can be recognized by DCs [104]. Furthermore, MOS supplementation has been shown significantly to increase antibody titers against Newcastle disease virus, thereby indicating a role of DCs in recognizing PAMP structures and initiating antibody response, which is true also in the case of AIV [105,106,107,108]. In a study by Gomez-Verdusco et al. [108], MOS supplementation significantly increased IgA production in the intestine and subsequently significantly decreased *Eimeria* oocyst output in chickens. It is noteworthy that results from studies with MOS supplementation suggest an ability of MOS to induce a Th1-type immune response. Some experiments have observed increased production of immunoglobulins associated with the Th2 pathway. Further research focused on DCs is needed to distinguish which factors influence the immunomodulatory effects of MOS.

### 6.2. β-Glucans

β-glucans are composed by polymerization of glucose through 1,3/1,6 β-glycoside linkages [109]. In addition to their occurring in the yeast cell wall, we can find β-glucans in other sources having different structures. For instance, β-glucans in cereals have 1,4 β-linkages between the glucopyranosyl molecules. β-glucans located in bacteria cell wall have only 1,3 β-linkages [110].

Many studies have been performed to determine β-glucans’ immunomodulatory properties in chicken intestines. Experiments have proven the binding capacity of barley β-D-glucan to the dectin-1 receptor [111,112]. Additionally, stimulation of dectin-1 with the specific ligand curdlan caused significant increase in production of reactive oxygen species in chicken peripheral blood mononuclear cells [113]. In several studies, it has been proven that addition of β-glucans to chicken diets led to strong up-regulation of nitric oxide synthase [114,115]. DC activation is accompanied by an increase in nitric oxide synthase production [31]. Based on findings described above, it appears that the main receptor involved in β-glucan recognition is dectin-1, a molecule with T cell stimulatory capacity [116]. In macrophages, Yadav and Schorey [117] showed that β-glucans were recognized by the dectin-1/TLR 2 receptor complex. β-glucans are well-known immunomodulators, and several studies have been conducted to determine which pathway (Th1 or Th2) is favored after β-glucan supplementation. Up-regulation of IL-2 and IL-18 expression had previously been described, thus suggesting involvement of the Th1 immune response [118,119]. Moreover, Cox et al. [114] described robust down-regulation of IL-4 and IL-13 gene expression in chicken intestines. In another study, Cox et al. [115] challenged chickens with *Eimeria* parasites and found protective effects of β-glucan supplementation through activation of the Th1 pathway, which is necessary for an appropriate immune response against *Eimeria*. In a study with mice, Ding et al. [116] reported an indispensable role of DC autophagy in activation of cytotoxic T cells by the Th1 pathway upon β-glucan activation. Interestingly, autophagy-deficient DCs showed down-regulation of costimulatory molecules CD80, CD86, and MHC II, as well as decreased production of TNF-α and nitric oxide synthase. CD4^+^ T cells cocultured with autophagy-deficient DCs had depressed IFN-γ production. Results of these studies support the view that β-glucans induce Th1 pathway activation. On the other hand, in studies by Cox et al. IFN-γ was down-regulated [114,115]. DCs produce IFN-γ in order to activate Th1 cells, and they produce additional IFN-γ and IL-2 for stimulating natural killer cells, macrophages, and cytotoxic T cells, as well as to inhibit the Th2 pathway [120]. Macrophages stimulated by β-glucans produce IL-1, a cytokine involved in the Th2 pathway. In the case of IL-4, which is a crucial cytokine for the Th2 pathway, inconsistent results were found. In *Eimeria*-challenged chickens fed with β-glucans, IL-4 was down-regulated [115], but if chickens were unchallenged with pathogens at 14 days of age, IL-4 was up-regulated in chickens fed supplemental β-glucan [114]. Additionally, chickens fed with β-glucans have slightly higher serum levels of IgG and much higher production of intestinal IgA [119]. Moreover, *salmonella*-treated chickens fed supplemental β-glucans had higher levels of serum IgG and IgA-producing cells in the intestine [121]. IgA can have immunomodulatory properties because it has been proven that IgA is involved in down-regulation of IFN-γ, TNF-α, and IL-6 while sustaining the level of IL-10. In this manner, IgA suppresses severe inflammation caused by some pathogens [122]. DCs play a key role in harmonizing inflammatory processes, and they possess the ability to produce IFN-γ, TNF-α, IL-6 [31], and IL-10 [123,124]. DC-SIGN and MR are involved in IgA recognition by DCs [125]. Recognition of IgA by DCs is accompanied by inhibition of the Th1 cytokine IL-12 [126] and increased IL-10 production [127]. Increased IgA production in the intestine may have a regulatory effect on intestinal DCs.

β-glucan particle size is an important factor for DC recognition by different receptors. Elder et al. [128] evaluated the influence of curdlan (a large β-glucan molecule) and curdlan microparticles (a small β-glucan molecule) on human DCs. They suggested critical roles of phagocytosis and dectin-1 receptor. Curdlan microparticles are phagocytosed easily, resulting in loss of dectin-1 surface expression. On the contrary, curdlan is not phagocytosed, and dectin-1 expression is maintained. Curdlan stimulated expression of IL-1β, IL-6, and IL-23 by DCs whereas curdlan microparticles did not stimulate IL-1β, IL-6, and IL-23 expression. TSLP and CCL22, factors associated with the Th2 immune response, were not influenced by β-glucan particle size. Therefore, DCs may recognize prebiotic particles by different receptors, and subsequently DCs produce cytokines in order to stimulate different pathways.

### 6.3. Short-Chain Fatty Acid Production Induced by Fructooligosaccharides

Fructooligosaccharides (FOSs) consist of as many as 10 monomeric β-2/1-linked fructosyl units. The best-known FOS is inulin, a widely used prebiotic obtained from chicory root [129]. Relatively consistent results have been observed in experiments evaluating effects of FOS supplementation on the immune response in poultry. For example, broilers supplemented with several levels of inulin expressed lower levels of IL-6 and IFN-γ and, conversely, significantly increased cecal IgA concentration [130]. FOS caused significantly higher numbers of IgA-positive cells in the lamina propria of the ileum and, at the same time, up-regulation of the important Th2 cytokine IL-4 in *salmonella*-challenged laying hens [131]. Additionally, FOS treatment has been shown to significantly increase IgG and IgM antibody titers in plasma [132]. These results suggest down-regulation of the Th1 response and up-regulation of the Th2 pathway in FOS-supplemented chickens.

FOSs have low degradability by intestine enzymes due to their β-2/1-linked fructosyl bonds, so large quantities of FOS pass into the ceca [133]. Short-chain fatty acids (SCFAs) are FOS metabolites produced by microbiota in chicken ceca, and some of them show anti-inflammatory properties that can explain the indirect influence of FOS on the gastrointestinal immune system [134]. Ding et al. [135] evaluated butyrate, propionate, valerate, and acetate production in chickens supplemented with FOS. They found increased production of all evaluated SCFAs, but the highest production was of butyrate. Similarly, Rehman et al. [133] found the highest production of butyrate and slightly greater production of propionate in inulin-fed chickens. On the contrary, levels of valerate and acetate were not affected by inulin treatment.

In an avian model, only Babu et al. [136] have tested the influence of FOS on the ability of chicken HD11 macrophages to phagocytose and eliminate *Salmonella enteritidis*. Their results showed improved ability of HD11 macrophages to eliminate *S. enteritidis* by preventing IL-1β-associated macrophage apoptosis. The direct influence of SCFAs on avian DCs is poorly understood, but mechanisms by which SCFAs affect DC function have been reported in other species. DCs and other immune cells express specific receptors for microbial metabolites. Nastasi et al. [137] described GPR43 and GPR109A receptors on human monocyte-derived DCs (HMDDCs). GPR43 is stimulated primarily by acetate and propionate, and GPR109A is a receptor for butyrate [138]. HMDDCs treated with butyrate and propionate down-regulated pro-inflammatory chemokines CCL3, CCL4, CCL5, CXCL9, CXCL10, and CXC. Moreover, butyrate and propionate inhibited expression of LPS-induced IL-6 and IL-12p40 cytokine production, yielding a strong anti-inflammatory effect on DCs [137]. In another study, butyrate inhibited the Th1 pathway by inhibiting IL-12 and IFN-γ and promoting IL-10 cytokine production in HMDDCs. Expressions of costimulatory molecules CD80, CD83, and MHC II were substantially reduced by butyrate [139]. SCFA concentration is known to play an important role, as Iraporda et al. [140] proved dose-dependent inhibition of pro-inflammatory cytokines by butyrate and propionate. Butyrate and propionate have shown to be effective from concentrations of 1–5 mM. Singh et al. [141] revealed the importance of GPR109A activation in DC-mediated immunomodulation caused by butyrate. GPR109a-knockout mice had reduced numbers of IL-10-producing CD4^+^ T cells and an increase in those of IL-17-producing T cells. Additionally, DCs incubated with butyrate or niacin (the next ligand of GPR109A) had higher expressions of l10 and Aldh1a1, factors involved in differentiation of naive T cells to regulatory T cells. FOS supplementation also increases the numbers of *Bifidobacterium* in chicken cecum [142]. Coculture of *Bifidobacterium* with monocyte-derived DCs demonstrated production of IL-2, an important cytokine for regulatory T cell expansion [143]. Based on these findings, Corrêa-Oliveira et al. [144] suggested DC-mediated activation of regulatory T cells in order to suppress inflammatory T cells after butyrate treatment. In the chicken genome, more than 20 paralogs of GPR43 were identified, and the strongest expression of GPR43 was found in peripheral blood mononuclear cells [145]. However, occurrence of the GPR109A receptor in chickens has never been studied [146]. SCFAs affect DC function, and similar modes of action can be assumed in chickens as in mammals.

### 6.4. Chitosan Oligosaccharides

Chitosan, an insoluble material that is not broken down by digestive enzymes, consists of N-acetylglucosamine units with β-1/4 linkages [147]. DCs can recognize chitin fragments by several receptors, while MR ensures endocytosis and formation of endosomes. Dectin-1 activation induces phagocytosis and respiratory burst. TLR 2 signaling activation causes up-regulation of pro-inflammatory cytokines such as IL-12 and TNF-α [148]. Huang et al. [147] observed increased levels of plasma IgG, IgM, and IgA in chitosan-fed chickens. In another study, increased levels of IgM were found, but there was no effect on IgG and IgA. Additionally, higher serum concentrations of IL-1β, IL-6, and IgM, as well as greater nitric oxide synthase activity, were determined in chickens 21 days old that were fed chitosan. Older (42 days) chickens had higher levels of TNF-α and IFN-γ [149]. It has been proven that differences in chitosan fragment size are associated with up-regulation of different receptors. C-type lectins, dectin-1, and MR seem to be up-regulated by smaller particles under 40 μm. TLR 2 recognizes particles between 40–70 μm and subsequently stimulates pro-inflammatory pathways. For this reason, chitosan can modulate production of cytokines by stimulating different DC receptors [150].

## 7. Conclusions and Future Developments

Better understanding of the interactions between avian DCs and prebiotics has huge potential for creating more effective prebiotic feed additives or vaccine adjuvants in poultry. We have no information to date about the effects of galactooligosaccharides and xylooligosaccharides on DC function, so further research is needed in those areas. Moreover, it is necessary to determine the direct or indirect ways that prebiotics can influence DC function to modulate the immune response. In the gut, prebiotics can change microbiota composition as well as stimulate production of SCFAs and other substances that could influence DC. That means it is difficult to recognize which factor is involved in DC-induced immunomodulation. For this purpose, numerous in vitro studies must be performed to determine impacts of prebiotics on gastrointestinal DCs. Nowadays, there is a methodology to culture DCs from precursor cells in bone marrow. Nevertheless, because DCs located in the intestine express some unique properties, and for the sake of more objective results, it would be ideal to culture these cells from the intestine. A future development in DC culture would be associated with the use of 3D cell cultures. This approach promises to culture DCs directly from gastrointestinal-associated lymphoid tissue and for subsequent use in in vitro studies.
